# Dietary intake patterns among adults with inflammatory bowel disease in the United States, 2015

**DOI:** 10.1371/journal.pone.0250441

**Published:** 2021-04-21

**Authors:** Fang Xu, Sohyun Park, Yong Liu, Kurt J. Greenlund

**Affiliations:** 1 Division of Population Health, National Center for Chronic Disease Prevention and Health Promotion, Centers for Disease Control and Prevention, Atlanta, Georgia, United States of America; 2 Division of Nutrition, Physical Activity, and Obesity, National Center for Chronic Disease Prevention and Health Promotion, Centers for Disease Control and Prevention, Atlanta, Georgia, United States of America; The University of Hong Kong, HONG KONG

## Abstract

**Background:**

Dietary behavior and nutrient intake patterns among U.S. men and women with inflammatory bowel disease (IBD) are unclear at the population level.

**Methods:**

This cross-sectional study compared dietary intake patterns among U.S. adults (aged ≥18 years) with and without IBD in the 2015 National Health Interview Survey (N = 33,626). Age-standardized weighted prevalences for intake of fruits, vegetables, dairy, whole grain bread, dietary fiber, calcium, total added sugars, sugar-sweetened beverages (SSBs), processed meat, and supplement use were compared between adults with and without IBD by sex.

**Results:**

In 2015, an estimated 3 million adults (1.3%) reported IBD. Compared with adults without IBD, adults with IBD were more likely to be older, non-Hispanic white, not currently working, former smokers, and former alcohol drinkers. Overall, dietary behaviors were similar among adults with and without IBD. However, adults with IBD were more likely to take vitamin D supplements (31.5% vs 18.8%) and consume dietary fiber <16.7 grams(g)/day, the amount that 50% of U.S. adults consumed (51.8% vs 44.1%), than those without IBD. Compared with their counterparts, men with IBD were more likely to consume vegetables ≥1 time/day (84.9% vs 76.0%) and take any supplement (59.6% vs 46.0%); women with IBD were more likely to have SSBs ≥2 times/day (26.8% vs 17.8%) and total added sugars ≥14.6 teaspoons(tsp)/day, the amount that 50% of U.S. adults consumed (55.3% vs 46.7%).

**Conclusions:**

Adopting a healthy diet, especially limiting added sugars intake among women with IBD, might be important for the overall health.

## Introduction

Inflammatory bowel disease (IBD), encompassing Crohn’s disease and ulcerative colitis, is characterized by chronic inflammation that occurs in the large intestine and rectum in ulcerative colitis and any part of the gastrointestinal tract in Crohn’s disease including the small intestine [1]. The number of U.S. adults having received a diagnosis of IBD considerably increased from 1.8 million in 1999 [2] to 3 million in 2015 [3]. Globally, the number of cases of IBD has increased from 3.7 million in 1990 to 6.8 million in 2017, which could cause substantial burden on healthcare costs worldwide [4].

Nutrition is an important aspect for IBD care. Although the mechanism is not well understood, diet along with other environmental factors are believed to have an impact on the intestinal microbiome which may trigger inflammation [5]. Malnutrition is prevalent among patients with IBD, with the prevalence varying from 18% to 75% and is higher in patients with Crohn’s disease than patients with ulcerative colitis [6], which could lead to emergency department visits and hospitalizations [7]. Several dietary interventions, such as specific carbohydrate diet, anti-inflammatory diet, and Mediterranean diet, have been suggested to patients with IBD depending on their symptoms or medications [5]. The European Society for Clinical Nutrition and Metabolism recently provided a guideline of nutritional supports to patients with IBD at various disease stages [8]. To date, however, there is no universally effective dietary therapy to IBD patients, or an IBD-specific diet during active disease and remission phases [9]. Patients with IBD usually hold their own dietary beliefs as to what food they take may relieve digestive symptoms [10].

Information about the pattern of dietary intake and certain nutrient intake at the population level is limited, and dietary behaviors might differ by sex [11]. Therefore, this study sought to assess dietary intake behaviors among community-dwelling U.S. adult men and women with and without IBD in a national survey.

## Materials and methods

### Data source

The 2015 National Health Interview Survey (NHIS), conducted by the National Center for Health Statistics, is a nationally representative cross-sectional household interview survey of the civilian noninstitutionalized population [12]. The survey includes a broad range of health topics including sociodemographic characteristics, chronic diseases, health-risk behaviors, and healthcare access. In 2015, the Sample Adult File (response rate of 55.2%) included a question about IBD status, and the Cancer Control Supplement File contained questions about diet and nutrition asked to all adults included in the Sample Adult File.

### Definition of IBD, dietary and nutrient intake

IBD was defined as an affirmative response to the question, “Have you ever been told by a doctor or other health professional that you had Crohn’s disease or ulcerative colitis?” The survey questions asked about dietary intake during the past month and each of the food and drink items was measured in frequency. Fruits (<1 or ≥1 time/day) included drinking 100% pure fruit juice or fresh, frozen, or canned fruits. Vegetables (<1 or ≥1 time/day) included green leafy or lettuce salads, any kind of fried potatoes, or other kind of potatoes, and all other vegetables excluding lettuce salads, potatoes, and cooked dried beans. We also measured vegetable consumption excluding any kind of fried potatoes. Dairy products (<1 or ≥1 time/day) included milk either to drink or on cereal excluding soymilk or small amounts of milk in coffee or tea, and any kind of cheese excluding cheese on pizza. Whole grain bread (<1 or ≥1 time/day) included wheat, rye, oatmeal, and pumpernickel on toast, roll and in sandwiches. Sugar-sweetened beverages (SSBs, <2 or ≥2 times/day) was measured according to frequency of drinking sports and energy drinks, coffee or tea with sugar or honey added to it, sweetened fruit drinks, and regular soda or pop containing sugar. Processed meat (<1 or ≥1 time/week) included bacon, lunch meats or hot dogs. To measure certain nutrient intakes including dietary fiber, calcium, and total added sugars that were assessed in this study, we used an algorithm developed by the National Cancer Institute (NCI) [13]. The Dietary Screener Questionnaire (DSQ), originally developed for the 2009–2010 National Health and Nutrition Examination Survey (NHANES), included food and drink items such as vegetables, fruits, whole grain, and SSBs with frequency responses. Based on the 24-hour recall data from the NHANES, frequencies of food and drink items were adjusted by age- and sex-specific median portion size, and a regression model was used to predict the nutrient intake [14]. The DSQ administered in the 2015 NHIS diet and nutrition measures was almost identical to that in the 2009–2010 NHANES. The DSQ scoring algorithm was validated and is a useful tool when an assessment of total dietary intake was unavailable [14]. Calcium intake was categorized as <1000 or ≥1000 milligrams (mg)/day [15]. The recommended Adequate Intake (AI) of total fiber ranges from 21 grams to 38 grams per day based on age and sex [16]. However, the NHIS data only estimated dietary fiber intake, not total fiber intake. Furthermore, because of a small number of adults reporting AI levels, estimates comparing the recommended levels of fiber intake by IBD status were not reliable. We therefore categorized dietary fiber intake based on the median (16.7 g/day), the amount that 50% U.S. adults consumed daily, which was close to the average dietary fiber intake (17.0 g/day) among adults aged ≥20 years in the 2009–2010 NHANES [17]. Percentages of dietary fiber intake below the median were estimated and compared by IBD status. Total added sugars were also categorized at the median, but percentages of total added sugars above median (≥14.6 or <14.6 tsp/day) were estimated and compared by IBD status. Supplements included any supplement, multivitamins, calcium, and vitamin D taken during the past month.

### Sociodemographic and health-related variables

Sociodemographic characteristics included age group (18–44, 45–64, or ≥65 years), sex, Hispanic origin and race (non-Hispanic white, non-Hispanic black, Hispanic, or non-Hispanic other), and education level (less than high school, GED or high school graduate, some college, or bachelor’s degree or higher). Marital status was categorized as “married or living with partner” or “not married” (combining widowed, divorced, separated, or never married). Current employment status during the week before the interview was defined as “working” if respondents were working for pay at a job or business, having a job or business but not at work, or working but not for pay at a family-owned job or business. “Not working/not looking” employment status included not working but looking for work, and not working and not looking for work. Based on the U.S. Census Bureau’s poverty thresholds, poverty status was determined using the NHIS’ imputed income file [18], including “poor” if family incomes were <100% of the federal poverty level (FPL), “near poor” if family incomes were ≥100% to <200% of the FPL, or “not poor” if family incomes were ≥200% of the FPL.

Health-related behaviors included weight status, smoking status, and alcohol drinking status. Weight status, measured as body mass index (BMI) based on self-reported weight and height, was categorized as underweight or normal weight (BMI<25 kg/m^2^), overweight (BMI ≥25 to <30 kg/m^2^), or obesity (BMI ≥30 kg/m^2^). Smoking status was categorized as never smoker, former smoker, or current smoker. Alcohol drinking status included lifetime abstainer (adults having <12 drinks in lifetime), former drinker (adults having had ≥12 drinks in lifetime but none in the past year), or current drinker (adults having had ≥12 drinks in lifetime and ≥1 drink in the past year).

### Statistical analysis

The sample adult weights were used in the analyses. Weighted percentages of demographic characteristics and health-related behaviors were calculated by IBD status. The prevalences of the dietary intake were calculated by IBD status and sex and were age-standardized to the 2000 U.S. Standard Population [19]. Group differences were determined by t-test by specifying the linear contrasts at the significance level of 0.05. Analyses were performed using SAS-callable SUDAAN to account for complex sampling design of the NHIS (11.0.3, Research Triangle Institute, North Carolina, US). The NHIS data are publicly available and the Institutional Review Board approval was not required.

## Results

In the 2015 NHIS, an estimated 3 million adults had IBD (1.3%). Compared with adults without IBD, adults with IBD were more likely to be older, non-Hispanic whites, not currently working, former smokers, and former alcohol drinkers, and less likely to be non-Hispanic blacks, never smokers, and current alcohol drinkers (Table 1). Adults with IBD were more likely to consume vegetables (including or excluding fried potatoes) ≥1 time/day, take vitamin D supplements, and consume dietary fiber less than 16.7 g/day, the amount that 50% of U.S. adults consumed, than those without IBD (Table 2). When stratified by sex, compared with their counterparts, men with IBD were more likely to consume vegetables ≥1 time/day and take any supplements whereas women with IBD were more likely to drink SSBs ≥2 times/day, and consume total added sugars more than 14.6 tsp/day, the amount that 50% of U.S. adults consumed.

**Table 1:** Distribution of sociodemographic characteristics and health-related behaviors among adults by inflammatory bowel disease status, United States, 2015.

Characteristics	Adults with IBD % (95% CI)	Adults without IBD % (95% CI)
**Unweighted N**	454	33,172
**Weighted N**^a^	3,087,000	239,025,000
**Age group (years)**		
18−44	33.0 (27.4−39.0)***	46.7 (45.8−47.6)
45−64	41.0 (35.1−47.1)*	34.2 (33.5−35.0)
≥65	26.1 (20.9−32.0)*	19.1 (18.4−19.7)
**Sex**		
Male	42.6 (36.4−49.1)	48.3 (47.5−49.0)
Female	57.4 (50.9−63.6)	51.7 (51.0−52.5)
**Race and Hispanic origin**		
White, non-Hispanic	76.0 (70.6−80.7)***	64.8 (63.9−65.6)
Black non-Hispanic	5.1 (3.2−7.8)***	11.8 (11.2−12.4)
Hispanic	12.8 (9.3−17.4)	15.6 (15.0−16.3)
Other^b^ or multirace, non-Hispanic	6.2 (3.6−10.4)	7.8 (7.4−8.2)
**Educational attainment**		
Less than high school	16.7 (12.0−22.9)	12.5 (12.0−13.1)
GED or high school graduate	25.1 (19.9−31.0)	24.8 (24.1−25.5)
Some college	30.7 (25.5−36.4)	31.2 (30.4−31.9)
University graduate	27.6 (22.4−33.4)	31.5 (30.6−32.4)
**Marital status**		
Married/living with partner	60.5 (54.5−66.2)	60.4 (59.6−61.2)
Not married	39.5 (33.8−45.5)	39.6 (38.8−40.4)
**Current employment status**		
Working	49.5 (42.9−56.1)***	61.6 (60.8−62.4)
Not working/not looking	50.5 (43.9−57.1)***	38.4 (37.6−39.2)
**Poverty status**^c^		
Poor	16.1 (12.2−20.9)	12.5 (12.0−13.1)
Near poor	17.9 (13.1−23.9)	18.5 (17.8−19.2)
Not poor	66.1 (59.9−71.8)	68.9 (68.1−69.9)
**Weight status (BMI, kg/m**^**2**^**)**		
Underweight/normal weight (<25)	36.8 (30.8−43.3)	36.2 (35.4−37.0)
Overweight (25−<30)	36.9 (31.4−42.7)	33.7 (33.0−34.4)
Obesity (≥30)	26.3 (21.2−32.1)	30.0 (29.3−30.8)
**Smoking status**		
Never smoker	53.5 (47.9−59.0)***	63.2 (62.4−63.9)
Former smoker	30.5 (25.2−36.5)**	21.7 (21.1−22.4)
Current smoker	16.0 (11.9−21.0)	15.1 (14.6−15.7)
**Alcohol drinking status**		
Lifetime abstainer	16.3 (12.1−21.6)	20.8 (20.0−21.5)
Former drinker	25.9 (20.8−31.7)***	14.1 (13.6−14.7)
Current drinker	57.8 (51.6−63.7)*	65.1 (64.3−66.0)

Abbreviations: IBD, inflammatory bowel disease; CI, confidence interval; GED, General Education Diploma; BMI, body mass index.

*P<0.05

**P<0.01

***P<0.001.

^a^Estimated number rounded to 1,000s.

^b^Other includes American Indians or Alaskan Natives and Asians or Pacific Islanders.

^c^Poverty status was derived based on the imputed income file.

**Table 2:** Age-standardized^a^ prevalence of dietary intake among adults with or without inflammatory bowel disease by sex, United States, 2015.

Dietary or supplement intake during the past month	All adults (N = 33,626)		Men (n = 15,051)		Women (n = 18,575)	
	With IBD (n = 454) % (95% CI)	Without IBD (n = 33,172) % (95% CI)	With IBD (n = 163) % (95% CI)	Without IBD (n = 14,888) % (95% CI)	With IBD (n = 291) % (95% CI)	Without IBD (n = 18,284) % (95% CI)
**Dietary intake**^b^						
Fruits (≥1 time/day)^c^	58.0 (50.4–65.3)	56.6 (55.7–57.4)	54.0 (41.3–66.1)	52.7 (51.5–53.9)	61.2 (51.7–70.0)	60.2 (59.1–61.3)
Vegetables (≥1 time/day)^d^	84.9 (80.2–88.6)**	78.8 (78.2–79.4)	84.9 (77.6–90.1)**	76.0 (75.0–77.0)	84.8 (78.1–89.7)	81.5 (80.7–82.3)
Vegetables excluding fried potatoes (≥1 time/day)	77.2 (71.3–82.3)*	71.2 (70.4–71.9)	75.2 (65.0–83.2)	66.8 (65.6–67.9)	78.8 (71.3–84.7)	75.4 (74.4–76.3)
Dairy products (≥1 time/day)^e^	64.6 (57.6–71.0)	59.9 (59.1–60.6)	65.5 (55.3–74.5)	61.0 (59.9–62.1)	64.2 (54.5–72.9)	58.8 (57.8–59.7)
Whole grain bread (≥1 time/day)^f^	27.9 (21.9–34.8)	28.6 (27.9–29.4)	32.1 (22.9–43.0)	29.1 (28.1–30.1)	24.5 (17.6–33.0)	28.2 (27.3–29.2)
Dietary fiber (<16.7 g/day)^g^^,^^h^	51.8 (44.7–58.7)*	44.1 (43.3–44.9)	31.3 (21.7–42.8)	23.8 (22.9–24.7)	67.2 (59.1–74.5)	63.2 (62.1–64.3)
Calcium (≥1000 mg/day)^g^^,^^i^	43.9 (37.1–50.9)	50.1 (49.3–50.9)	72.2 (62.3–80.3)	77.7 (76.8–78.6)	22.7 (15.4–32.2)	24.2 (23.3–25.1)
Total added sugars (≥14.6 tsp/day)^g^^,^^h^	63.0 (56.4–69.1)	57.8 (57.0–58.5)	73.2 (62.8–81.6)	69.5 (68.5–70.5)	55.3 (46.7–63.5)*	46.7 (45.7–47.8)
Sugar-sweetened beverages (≥2 times/day)^j^	27.9 (21.4–35.5)	21.1 (20.5–21.8)	29.3 (18.2–43.5)	24.6 (23.6–25.7)	26.8 (19.7–35.3)*	17.8 (17.0–18.7)
Processed meat (≥1 time/week)^k^	41.0 (34.4–47.9)	45.4 (44.5–46.2)	36.2 (27.1–46.4)	40.4 (39.3–41.6)	44.6 (35.6–53.9)	50.0 (49.0–51.0)
**Supplement intake**						
Any^l^	60.7 (52.6–68.3)	52.9 (52.1–53.8)	59.6 (46.8–71.3)*	46.0 (44.8–47.2)	61.2 (51.2–70.4)	59.5 (58.4–60.6)
Multivitamin	40.7 (33.5–48.2)	37.7 (36.9–38.5)	45.5 (33.6–57.9)	33.2 (32.1–34.3)	37.4 (29.5–46.1)	42.0 (40.9–43.1)
Calcium	18.0 (14.3–22.4)	15.2 (14.7–15.7)	—^m^	—^m^	25.9 (20.4–32.2)	21.2 (20.4–22.1)
Vitamin D	31.5 (25.2–38.6)***	18.8 (18.2–19.5)	23.6 (17.1–31.7)**	13.7 (12.9–14.5)	37.2 (27.9–47.5)**	23.6 (22.8–24.5)

Abbreviations: IBD, inflammatory bowel disease; CI, confidence interval; g, gram; mg, milligram; tsp, teaspoon.

*P<0.05

**P<0.01

***P<0.001.

^a^According to the 2000 U.S. standard population and three age groups: 18–44, 45–64 and ≥65 years.

^b^Based on the data distribution, the cutoff of 1 time/day was determined for fruits, vegetables, dairy products, whole grain bread, and 1 time/week was determined for processed meat.

^c^Included drinking 100% pure fruit juice such as orange, mango, apple, grape and pineapple juices or fruits including fresh, frozen, or canned fruit.

^d^Included green leafy or lettuce salads, any kind of fried potatoes including French fries, home fries, or hash brown potatoes, other kind of potatoes such as baked, boiled, mashed potatoes, sweet potatoes, or potato salad, and all other vegetables excluding lettuce salads, potatoes, and cooked dried beans.

^e^Included milk either to drink or on cereal excluding soymilk or small amounts of milk in coffee or tea, and any kind of cheese including that as a snack, on burgers, sandwiches, and in foods such as lasagna, quesadillas, or casseroles, but excludes cheese on pizza.

^f^Included whole wheat, rye, oatmeal, and pumpernickel on toast, roll and in sandwiches.

^g^The measures of dietary fiber, calcium, and total added sugars used the score algorithm based on Dietary Screener Questionnaire developed by the National Cancer Institute.

^h^The cutoff was based on median as the data was not normally distributed.

^i^According to the Institute of Medicine Committee to Review Dietary Reference Intakes for Vitamin D and Calcium, Food and Nutrition Board, the recommended average required doses for calcium intake for adults are 800 mg, 1000 mg, and 1200 mg based on age and sex. In this study, 1000 mg was used as the cutoff.

^j^Measured based on 4 questions: 1. How often did you drink sports and energy drinks such as Gatorade, Red Bull, and Vitamin water? 2. How often did you drink coffee or tea that had sugar or honey added to it? Include coffee and tea you sweetened yourself and presweetened tea and coffee drinks such as Arizona Iced Tea and Frappuccino. Do not include artificially sweetened coffee or diet tea. 3. How often did you drink sweetened fruit drinks, such as Kool-aid, cranberry and lemonade? Include fruit drinks you made at home and added sugar to. 4. How often did you drink regular soda or pop that contains sugar? Do not include diet soda.

^k^Measured based on the question, “How often did you eat processed meat, such as bacon, lunch meats, or hot dogs?”

^l^Included any vitamin or mineral pills, or any supplements. If respondents answered yes to intake of any supplement question, they answered questions related to intake of multivitamin, calcium, and vitamin D.

^m^Data were suppressed because the relative standard errors were greater than 30%.

## Discussion

The present study, based on an assessment of selected dietary and nutrient intake, showed an overall similar dietary pattern at the population level between men and women with and without IBD. In addition to promotion of overall health, maintaining well-balanced nutrition is critical for IBD management. Studies have shown that IBD patients tend to avoid certain food which they believe can trigger symptoms and modify their diet on the basis of symptoms [10, 20–24]. However, self-imposed food restriction might be associated with malnutrition which could cause weakened muscle strength among some IBD patients [20]. There is no universal recommendation regarding which food consumption should be limited. Clinicians may have some dietary recommendations for patients with IBD to relieve symptoms but could have different opinions from their own clinical perspectives [23].

Although most dietary patterns were similar, the study showed some distinct dietary behaviors by IBD status. For instance, men with IBD were more likely to consume vegetables than those without IBD, but this association was not significant when assessing vegetable consumption excluding fried potatoes. Fried food usually contains more fat and calories than the same amount of non-fried food. Another example is that men and women with IBD were more likely to take vitamin D supplements. Although it is unknown whether a lower level of vitamin D would be a risk factor for developing IBD or the disease causes deficiency of vitamin D, prevalence of vitamin D deficiency is pronouncedly higher among IBD patients. In a multicenter prospective study, vitamin D deficiency was found in 78% of well-nourished and 90% of malnourished IBD patients [20]. A lower level of vitamin D is also associated with disease severity, infections, and risk for surgery and hospitalization [25]. Besides vitamin D, IBD patients are susceptible for inadequate levels of other vitamins and minerals [25]. In the current study, men but not women with IBD were more likely to report any supplemental intake than those without IBD.

We were not able to examine the difference of the recommended AI of total fiber [16] by IBD status because very few people consumed at these levels. Based on the 2015 NHIS data, 50% of U.S. adults consumed less than 16.7 grams of dietary fiber every day, which was far below the recommended levels [16]. Adults with IBD, however, were even more likely to have dietary fiber intake at these low levels compared with those without IBD. Patients with IBD were observed to have a low-fiber diet overall regardless of their disease activity [22], possibly because of the concerns of worsened symptoms [26]. Fibers might be avoided for IBD patients with strictures because of the obstructive symptoms [27]. However, fiber intake has health and clinical benefits and should not be restricted among patients with IBD [26]. Avoiding dietary fiber was actually found to be associated with a higher risk of flare-up in patients with Crohn’s disease [28]. Furthermore, low-fat and high-fiber diet was found to reduce inflammation and improved quality of life among patients with ulcerative colitis [29].

Noticeably, the current study showed that the prevalence of added sugars and SSBs was higher among women with IBD than among women without IBD. Although a previous study suggested that sugary foods might not worsen IBD symptoms compared with other trigger foods [21], some evidence has shown that high sugar consumption may be a risk factor for developing IBD because sugar might change the microbiome and cause inflammatory response [26]. A large European nested case and control study found that a diet of high sugar and soft drink especially with a low vegetable intake increased the risk of ulcerative colitis [30]. The dietary guidelines for the general U.S. population suggest that added sugars intake should be limited to <10% of daily total calorie intake [31]. Furthermore, excessive sugar intake may be linked with various chronic conditions in the general population such as diabetes, heart disease, and metabolic syndrome, and therefore should be prevented [32].

The study has several limitations. First, the NHIS does not include a comprehensive dietary and nutrition assessment. Several dietary intakes assessed in the current study used frequency, not amount, and energy intake was not available in the survey data. Nonetheless, we were able to transform some frequency measures into portion size unit by using the DSQ scoring algorithm, although it has a limited number of nutrient measures. Body weight and physical activity that ideally should be adjusted for energy intake [33] were not included in the scoring algorithm. However, the predicted nutrient intake took age, sex, and portion size into account. Second, the survey question was not designed for adults with IBD, so some detailed nutrition measures that are important to IBD such as types of fiber, omega fatty acids, and essential vitamins and minerals (e.g., zinc, magnesium, potassium) [34] could not be assessed. Third, Crohn’s disease was not differentiated from ulcerative colitis to assess whether dietary patterns were different by disease type. Fourth, IBD status was based on self-report rather than medical records and may be subject to reporting biases. Finally, although the study covers noninstitutionalized adults aged ≥18 years, the findings cannot be generalized to other populations including people on active duty with Armed Forces or those living in long-term care facilities, prisons, or foreign countries. Despite these limitations, this is the first study to assess some important dietary intakes among U.S. men and women with IBD at the national level. A strength of the study was to be able to measure certain nutrients by using a validated scoring algorithm.

## Conclusions

Future studies need to confirm the higher prevalence of added sugars among women with IBD than those without IBD. Diets that are high in sugars but low in vegetables and fruits are risk factors for noncommunicable diseases [35]. Currently, healthy food such as fresh vegetables and fruits, and omega-3 rich food is the specific dietary components recommended to IBD patients [36]. While ongoing clinical trials are investigating the mechanisms of nutrition in IBD [26], adopting a healthy well-balanced diet including limiting added sugars might be important for overall health among IBD patients.
